# DoloTest in General Practice Study: Sensitivity and Specificity Screening for Depression

**DOI:** 10.1155/2012/472505

**Published:** 2012-12-06

**Authors:** Kim Kristiansen, Pernille Lyngholm-Kjaerby, Claus Moe

**Affiliations:** ^1^EvidenceProfile, Engsoeparken 91, 7200 Grindsted, Denmark; ^2^Norpharma A/S, Slotsmarken 15, 2970 Hoersholm, Denmark; ^3^Geriatric Department, Bispebjerg University Hospital, Bispebjerg, 2400 Copenhagen, Denmark

## Abstract

*Background*. Coexistence of pain and depression has significant impact on the patient's quality of life and treatment outcome. DoloTest is a pain and HRQoL assessment tool developed to provide shared understanding between the clinician and the patient of the condition by a visual profile. *Aim*. To find the sensitivity and specificity of DoloTest as a screening tool for depression for patients in primary care. *Methods*. All patients coming to a primary care clinic were asked to fill in a DoloTest and a Major Depression Inventory. *Results*. 715 (68.5%) of 1044 patients entered the study. 34.4% came due to pain. 16.1% met depression criteria, and 26.8% of patients coming due to pain met criteria for depression. 65.6% of the men and 54.2% of the women meeting the criteria for depression came due to pain. Depressed patients had statistically significant higher scores on all DoloTest domains. Selecting the cutoff value for the domain “low spirits” to be “65” (0–100) for depression gave a sensitivity of 78% (70–85%) and a specificity of 95% (93–96%) for meeting depression criteria. *Conclusion*. DoloTest can with a high sensitivity and specificity identify persons meeting criteria for depression and is an easy-to-use screening tool to identify patients with the coexistence of pain and depression.

## 1. Introduction

Chronic pain and depression are among the most common health problems reported by patients attending primary care [1–3]. Diagnosing pain and its coexistence with depression and getting an overview of the pain patient's situation can be challenging and can have great consequences for the treatment outcome. Numerous studies have found pain associated with a significant limitation in daily activities, physical activities and with poor self-rated health [3, 4]. Pain is also associated with increased prevalence of depressive disorders [5, 6]. Studies from primary care have demonstrated that 22–40% of all contacts to primary care are due to pain [7]. Persistent pain is often comorbid with depression both in general pain conditions [4, 8] and in disease-specific conditions [9]. Though health care providers are advised to pay special attention to pain symptoms in patients with depression [10], studies have demonstrated that primary care physicians tend to associate pain with depression to a significant lesser extent than other somatic symptoms [10], although pain is the most prevalent somatic symptom in patients with depression. This tendency to underestimate the intensity of burden has also been proven for the symptom pain with significant differences between the patient's and the healthcare provider's grading of the intensity of the pain [11, 12]. 

**Figure 1:**
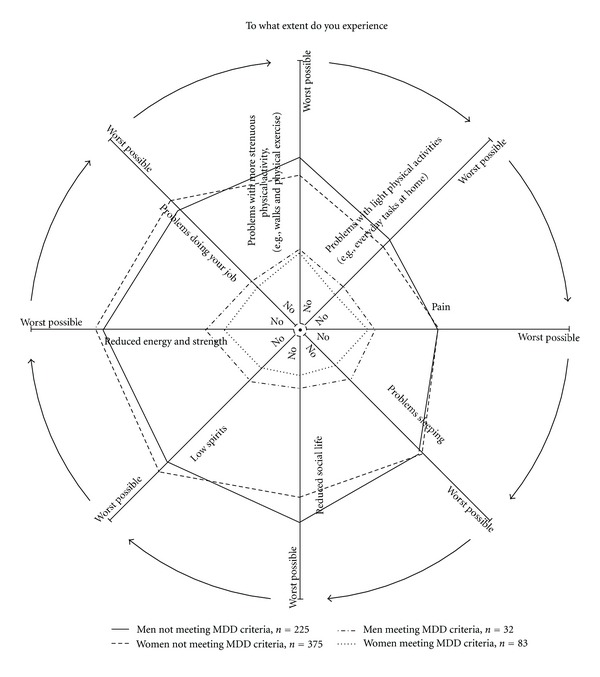
Average DoloTest-Profiles for men and women meeting criteria for depression (MDD) and not meeting criteria for MDD confidence interval in Table 3.

Patients with depression seek care more often for somatic symptoms than for psychological symptoms [13], why care providers must have an increased awareness for this coexistence. There is evidence that optimizing antidepressant therapy and pain self-management in primary care patients may result in improvement of both depression and pain [14]. Coexistence of depression and pain has also shown to be a major risk factor for opioid misuse both by using opioids for stress and sleep problems instead of for pain alone, and by using more opioids than prescribed [15]. It is therefore important that healthcare provider and the patient have an ongoing awareness and understanding of this connection between depression and pain and preferably share understanding of the current state of the condition. DoloTest (Figure 1) has been developed to provide such a shared understanding of the patients situation. It is a validated [16], pain- and health-related quality of life (HRQoL) assessment tool integrating measurement of the intensity of pain with assessment of the intensity of the impact of the pain including mood. 

The aim of this study was to determine to which degree DoloTest is useful as a screening tool for depression in primary care for adult (all persons 18 years or older) seeking primary care, and to find the sensitivity and specificity for DoloTest used for screening for depression.

## 2. Methods

The study is a diagnostic study among the population of patients visiting primary care physician no matter the reason for the visit. The study was conducted in the primary care clinic “Laegehuset NoerreTorv” in Grindsted, Denmark in the period from October 1st 2008 to December 31st, 2008. The clinic is a mixed urban and rural clinic with primary care physicians. The clinic serves approximately 6600 patients.

### 2.1. Participants

Denmark has open access to primary care free of charge. All patients being 18 years old or older visiting the clinic and not complying with the exclusion criteria were asked to fill in the questionnaires: DoloTest [16] and “Major Depression Inventory” (MDI) [17], both Danish versions. The study included a study population of 715 patients equal to a response rate of 68,5%. When arriving at the clinic, a clinic nurse screened for inclusion/exclusion criteria. Patients who could enter the study were handed the questionnaires together with a letter of introduction and the standardized DoloTest patient introduction [16]. Patients were also asked about age and gender and whether their visit was due to a pain problem or not. Patients were excluded if they meet exclusion criteria: cognitive dysfunction, visual impairment, and patients whose physical condition made filling the test impossible or prior participation in the study. 

### 2.2. Measurements and Variables

DoloTest is a validated pain- and health related quality of life (HRQoL) assessment tool integrating measurement of the intensity of pain with assessment of the intensity of the impact of the pain on eight 100 mm visual analogue scales (VAS). The design of DoloTest provides a presentation of the test result as a visual profile (DoloTest-Profile), with “no problems” toward the centre of each VAS-line and “worst possible” toward the periphery. The larger the DoloTest-Profile the worse the HRQoL, which is immediately understandable for both the patient and the healthcare provider [16]. The DoloTest-Profile enables the patient to take part in the evaluation of the test result and improve communication about the situation including problems related to depression like mood, sleep problems, tiredness functional problems, and allows the patient and the healthcare provider to evaluate response to treatment together. DoloTest is covering eight important aspects of HRQoL (Table 2). The test takes less than 2 minutes to complete [16]. The test results are also available as a DoloTest-Score, the sum of all scored domains, ranging from 0 to 800. DoloTest was filled in with one week recall.

 MDI is a validated self-assessment questionnaire developed to cover the diagnostic criteria in WHO's ICD-10 classification of major depressive disorders [18]. MDI was chosen because it follows the ICD-10 criteria and is easy to use as a self-assessment questionnaire. The MDI can be used both as a measuring instrument and as a diagnostic instrument with algorithms leading to the ICD-10 categories of depression. When used as a diagnostic instrument like in this study, the MDI items are dichotomized to indicate the presence or absence of each of the symptoms.

### 2.3. Variables

Data comes from 715 consecutive patients coming to primary care, no matter the reason for their visit. Scores on each DoloTest domain (Table 2) measured in millimetres. DoloTest Score is the sum of the measurement on each DoloTest domain in millimetres [16]. Depression was diagnosed according to the instruction for the MDI test [17].

### 2.4. Statistical Analysis and Ethics

Statistical analysis was made using Mann-Whitney test to compare groups and Chi-square test to compare frequencies using Analyse-It software v. 2.12. *P* < 0.05 was considered significant. Sensitivity/specificity was found using ROC curve analysis.

The study was according to Danish law ethical approved by “the Danish Data Protection Agency”, Copenhagen, Denmark, and all patients participated voluntary and were provided with both oral and written information.

## 3. Results

### 3.1. Study Population

A total of 1044 patients were asked to participate. Of those, 302 (31,5%) were not included either because they did not want to participate or due to the exclusion criteria. 27 provided incomplete data. 715 patients (68.5%) were included in the study. Of the 715 patients, 458 (64.1%) were women and 257 (35.9%) men. There were no statistically significant difference between the gender distribution in the study group and the excluded group (*P* = 0.59). The average age in the study population was 45.7 years and in the excluded group 55.4 years (*P* < 0.0001).

### 3.2. DoloTest Scores

Table 1 shows that 246 patients (34.4%) stated their visit was due to a pain problem (Study group) and 469 patients (65.6%) came for other reasons than pain (control group).

The study and control groups were sociodemographic identical with no statistical significant difference between the gender and age distributions, 33.6% of the woman and 35.8% of the men came due to pain (*P* = 0.56). 

Table 2 shows that in all domains there are statistically significant difference (*P* < 0.0001) between the study group and for the control group. “*low spirits*” and “*pain*” were the only domains in study group with statistically significant differences between men and women, women having the highest score. In the total study population, the women in average scored statistically significantly higher than men in all domains except for “*pain*” and “*problems with more strenuous activity*”.  In the control group, women scored statistically significantly higher in al domains except “*pain*”, where the women also scored higher than men, however not statistically significant. The DoloTest-Score was statistically significantly higher for women in the whole study population and also for the control group. In the study population the women's DoloTest-Score had a tendency to be higher than the men's. 

### 3.3. Major Depressive Disorder and DoloTest Profiles

Table 1 shows the prevalence of patients with depression for the study and control groups. 

115 (16.1%) of all the patients met criteria for depression using the MDI-scale. Table 2 presents the prevalence of depression together with the average MDI-rating score for the whole study population as well as specified for the study group and for the control group. In the whole study population, 32 men (12.8%) and 83 women (18.1%) met the criteria for depression using the MDI Scale, and the corresponding prevalence for control group was 11 men (6.7%) and 38 women (12.5%) with statistically significant difference between genders in both groups (*P* = 0.05). In the study population, 21 men (22.8%) and 45 women (29.2%) met criteria for depression (*P* = 0.27). The prevalence of patients with depression in study group was statistically significantly higher than in the control group (*P* = 0.0002) for men and *P* < 0.0001 for women. 

For patients meeting criteria for depression and patients not meeting the depression criteria, there was a difference for all DoloTest domains (*P* < 0.0001) (Table 3). The domains were scored equally by men and women meeting criteria for depression due to the MDI Scale; however, for patients not meeting criteria for depression, women scored statistically significantly higher than men on five of the domains (Problems with light physical activity, reduced energy and strength, low spirits, reduced social life, and sleeping problems) (Table 3). The numbers in Table 3 are visualized by presented as average DoloTest Profiles in Figure 1.

### 3.4. Sensitivity and Specificity Screening for Depression

**Figure 2:**
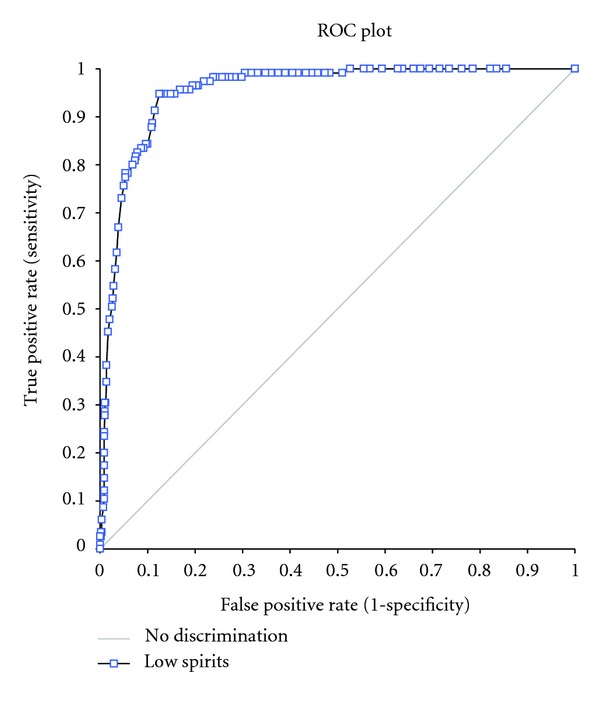


A cut-off value for the domain “*to what extent do you experience low spirits*” for depression was found using ROC curve analysis. It is desirable to find a cut-off with both a high sensitivity and a high specificity, and from a clinical point of view defining a cutoff with few false positive and high predictive value of positive test is important to help making an accurate clinical decision. Selecting a cutoff point of “65” (Figure 2) will provide a sensitivity of 0.78 (95% CI: 0.70–0.85) and a specificity of 0.95 (95% CI: 0.93–0.96) the predictive value of positive test being 0.74 for detecting that a patient meet criteria for depression. Area under the ROC-curve was 0.95 (95% CI: 0.94–0.97). 

## 4. Discussion

DoloTest has in this study proved useful as a screening tool for patients' experienced pain and the impact of pain on the patient's HRQoL as well as a screening tool for patients meeting criteria for depression and thereby for the coexistence of pain and depression. A cut-off value of “65” on the “low spirits” domain provides both high sensitivity (0.78) and high specificity (0.95) and a predicted value on positive test at 0.74.

This study is the first to present health related quality of life (HRQoL) data as DoloTest Profiles and DoloTest Scores for all patients seeking a primary care both for patients whose visit are due to a pain problem and for patients whose visit has other reasons. Furthermore, the study contributes to the growing knowledge about coexistence of pain and depression, finding the prevalence of depression to be statistically significantly higher for both genders in the group visiting primary care physician because of a pain problem than for other problems combined. 

### 4.1. Depression and Pain

Pain symptoms have been found to be associated with reduced psychological and physical health as well as with limitations in daily activities and with social isolation [19, 20] and are found to be a major reason for seeking health care [19, 20]. In this study, 34.4% of the patients stated that their visit to physician was due to pain, which is in accordance with findings in other studies [7].

Depression has shown to be present in 10–15% of all primary care patients [21]. Our study shows that 16.1% of all patients met criteria for depression, and 26.8% whose visit was due to pain, met depression criteria. 57.4% of patients who met the criteria for depression came due to pain. These findings are in good correlation with the literature, though this study captures the pain intensity during the last week, for example, not necessarily persistent pain.

The findings in this study are clinically important since the visual DoloTest Profiles can facilitate both awareness and often otherwise difficult communication about depression and pain, a coexistence leading to increased risk of reduced function, reduced work capacity, increased risk of opioid misuse, reduced HRQoL, and other related problems.

It has been shown that suffering from both pain and depression at the same time besides the low HRQoL is associated with dramatically increased health care costs [22, 23] compared to depression alone, and that treating depression alone does not give sufficient improvement on somatic symptoms like pain [21, 24]. Based on this knowledge, health care providers are encouraged to be aware of pain symptoms in patients with depression as well as depressive symptoms in patients with pain [21, 25]. Furthermore, it is stated that targeted screening for the cooccurrence of pain and depression is warranted [25]. The RESPECT trial [21] found that effective treatment of either depression or pain is dependent on the treatment of the other when they are co-existing. The study concludes “*it makes no longer sense to treat one condition without considering the other*” [21]. It is therefore of high importance to provide healthcare professionals with tools to screen and identify these patients at risk for suboptimal treatment if not found. 

### 4.2. Screening for Depression

In other studies using Visual Analogue Scale (VAS) as screening tool for depression, it has shown to be a reliable method [26] and VAS has thereby showed to be a valuable tool for assessment of mood [27]. A study looking at using one question from the Subjective Health Complaints (SHC) Inventory found this useful with sensitivity 79%, specificity 81% and predictive value of positive test to be 67% [28]. In the present study, DoloTest has proved as a tool screening both pain and depression, since the DoloTest domain “*low spirits*” is associated with depression. A cut off value of “65” has been found optimal for the DoloTest domain “*low spirits*” with a sensitivity of 78% and specificity of 95% to detect persons meeting criteria for depression, and with a predicted value on positive test at 0.74.

Pain is not included in neither the ICD-10 nor the DSM-IV criteria for depression, and it is also noteworthy that pain is not included in the most used tools for depression diagnostics like Hospital Anxiety and Depression Scale (HADS) and Beck Depression Inventory (BDI) [29] or in the Major Depression Inventory [17] (MDI) used in the present study, which increase the risk for not finding the coexistence. Both persistent pain and somatization have been found to be frequently associated with depression, yet few studies on somatization are controlled for depression [30]. 

### 4.3. Limitations and Strengths

Data is regarded representative for Denmark as the present study was conducted in a clinic serving both urban and rural areas. In Denmark, all citizens have free and equal access to primary care, as the primary care is financed through the Danish taxation system. The study has some limitations. The nature, diagnosis, or duration of pain were not recorded. All we asked for was experience in “the past week.” As expected the age distribution was statistically significantly higher in the excluded group due to the exclusion criteria cognitive and visual impairment. The scoring on both the DoloTest and MDI was made by the patients themselves and not controlled. Same physician made all diagnoses of meeting criteria for depression based on the MDI scorings; however, a depression diagnosis was not provided through interview or other further examination.

## 5. Conclusion

DoloTest used as a screening tool for depression criteria had a sensitivity of 78% and specificity of 95%, and a predicted value on positive test at 0.74. Patients meeting criteria for depression had statistically higher pain score and reduced Health Related Quality of Life than patients not meeting criteria for depression. 

DoloTest has in this study proved an easy-to-use screening tool in clinical settings for pain and the impact of pain as well as a screening tool for patient meeting criteria for depression. This ease the process of identifying patients with the important coexistence enabling easy identification of this large group of patients needing individualized treatment for more than the pain alone in order to get best possible health related quality of life. Treating both pain and depression when co-existing is very important since it otherwise is found to be associated with risk of reduced function, reduced work capacity, increased risk of opioid misuse, and increased healthcare costs.

## Figures and Tables

**Table 1:** Distribution of patients based on contact for the study and control groups and scores on DoloTest domain “*Pain*” for both gender for all patients and for patients with depression (MDD).

	All	Men total	Woman total	*P* m/w	Men MDD	Women MDD	*P* m/w
Study group	246/**34.4**%	92/**35.8**%	154/**33.6**%		21/**65.6**%	45/**54.2**%	0.26
	(30.9–37.9)	(29.9–41.7)	(29.3–38.0)	0.56	(49.2–82.1)	(43.5–64.9)	
Control group	469/**65.6**%	165/**64.2**%	304/**66.4**%		11/**34.4**%	38/**45.8**%	
	(62.1–69.1)	(58.3–70.1)	(59.6–68.4)		(17.9–50.8)	(35.1–56.5)	

Total	715	257	458		32	83	

**Table 2:** Average scores on DoloTest domains and DoloTest-Scores MDI Score and depression (MDD) prevalence ratio. *Statistical significance; *significant (*P* < 0.05).

	Contact: All			Study group			Control group			*P* value Pain/no pain
	Male	Female	*P* value	Male	Female	*P* value	Male	Female	*P* value	
	(*n* = 257)	(*n* = 458)	M/F	(*n* = 92)	(*n* = 154)	M/F	(*n* = 165)	(*n* = 304)	M/F	
Pain	28.0	31.6	0.085	48.4	54.0	0035*	16.6	20.2	0.059	<0.0001*
	(24.9–31.1)	(29.1–34.0)		(43.9–52.9)	(50.6–57.4)		(13.7–19.6)	(17.8–22.6)		
Problems with light physical activity	20.3	24.8	0.004*	33.8	38.9	0.070	12.8	17.7	0.002*	<0.0001*
	(17.4–23.2)	(22.6–27.1)		(28.3–39.2)	(34.9–42.9)		(10.0–15.5)	(15.4–20.0)		
Problems with more strenuous activity	31.5	33.0	0.28	50.9	49.6	0.86	20.6	24.5	0.025*	<0.0001*
	(27.7–35.3)	(30.2–35.7)		(44.8–57.0)	(44.8–54.5)		(16.6–24.6)	(21.5–27.4)		
Problems doing job	25.5	31.4	0.023*	45.3	43.3	0.21	18.2	25.3	0.006*	<0.0001*
	(21.6–29.3)	(28.3–34.1)		(39.3–50.9)	(39.0–47.6)		(13.9–22.4)	(21.9–28.7)		
	(*n* = 229)	(*n* = 415)		(*n* = 78)	(*n* = 127)		(*n* = 151)	(*n* = 288)		
Reduced energy and strength	32.6	40.8	<0.001*	47.6	53.7	0.065	24.4	34.2	<0.001*	<0.0001*
	(29.1–36.1)	(38.1–43.4)		(41.5–53.2)	(49.5–58.0)		(20.6–28.2)	(31.0–37.4)		
Low spirits	24.6	34.4	<0.0001*	34.6	43.3	0.008*	19.1	29.9	<0.0001*	<0.0001*
	(21.5–27.7)	(341.8–36.9)		(28.7–40.5)	(39.2–47.4)		(15.8–22.4)	(26.8–33.0)		
Reduced social life	22.0	26.6	0.002*	33.0	37.0	0.12	15.9	21.4	0.007*	<0.0001*
	(18.7–25.4)	(24.1–29.2)		(26.3–39.7)	(32.3–41.7)		(12.6–19.2)	(18.6–24.2)		
Problems sleeping	23.1	30.9	<0.001*	32.8	38.6	0.122	17.7	27.1	<0.001*	<0.0001*
	(20.0–27.8)	(28.3–33.6)		(26.8–38.9)	(34.0–43.2)		(14.4–21.3)	(24.0–30.2)		
DoloTest Score	204.8	250.6	<0.0001*	314.4	352.5	0.053	143.7	199.0	<0.0001*	<0.0001*
	(183.4–226.3)	(234.1–267.0)		(277.2–351.5)	(326.8–378.1)		(122.4–165.1)	(180.3–217.0)		
MDD prevalence ratio	0.128	0.181	0.048*	0.228	0.292	0.27	0.067	0.125	0.047*	<0.0001*
	(0.088–0.169)	(0.146–0.217)		(0.142–0.314)	(0.220–0.364)		(0.029–0.105)	(0.088–0.162)		

MDI: Major Depression Index; MDD: major depressive disorder; *significant (*P* < 0.05).

**Table 3:** Average score for all DoloTest domains and average DoloTest-Score for patients meeting criteria for depression (MDD) and patients not meeting MDD criteria.

	Patients not meeting MDD criteria			Patients meeting MDD criteria			Significance patients MDD/Not MDD	
	Male	Female	*P* value	Male	Female	*P* value	Male	Female
	(*n* = 225)	(*n* = 375)	M/F	(*n* = 32)	(*n* = 83)	M/F		
Pain	24.8	27.4	0.21	50.5	50.3	0.96	<0.0001*	<0.0001*
	(21.7–27.9)	(24.9–29.9)		(41.6–59.3)	(44.5–56.1)			
Problems with light physical activity	16.6	20.8	0.001*	46.0	42.9	0.69	<0.0001*	<0.0001*
	(14.0–19.3)	(18.6–23.1)		(35.7–56.3)	(37.5–48.3)			
Problems with more strenuous activity	27.0	28.1	0.36	62.9	55.0	0.22	<0.0001*	<0.0001*
	(23.3–30.7)	(25.3–30.6)		(52.0–73.8)	(48.2–61.8)			
Problems doing job	21.1	23.7	0.11	63.0	67.1	0.40	<0.0001*	<0.0001*
	(17.4–24.7)	(20.8–26.6)		(50.9–75.0)	(59.6–74.5)			
	(*n* = 205)	(*n* = 319)		(*n* = 24)	(*n* = 69)			
Reduced energy and strength	26.9	33.3	0.002*	72.7	74.4	0.58	<0.0001*	<0.0001*
	(23.7–30.1)	(30.7–35.9)		(65.9–79.4)	(70.4–78.4)			
Low spirits	18.4	25.7	<0.0001*	68.9	73.6	0.11	<0.0001*	<0.0001*
	(15.8–20.9)	(23.5–27.9)		(63.2–74.6)	(70.5–76.7)			
Reduced social life	14.9	19.3	0.002*	72.1	59.6	0.061	<0.0001*	<0.0001*
	(12.4–17.4)	(17.2–21.5)		(64.4–79.9)	(53.4–65.8)			
Problems sleeping	17.4	23.8	0.002*	62.9	63.3	0.76	<0.0001*	<0.0001*
	(14.8–20.1)	(21.3–26.2)		(54.3–71.5)	(57.9–68.8)			
DoloTest-Score	165.2	200.9	0.003*	483.2	474.9	0.77	<0.0001*	<0.0001*
	(146.9–183.5)	(185.7–216.1)		(431.5–534.9)	(448.5–501.3)			

*Statistical significance. Data presented visually in Figure 1.
